# Gastrointestinal endoscopic image style transfer using EndoStyle to improve artificial intelligence prediction models

**DOI:** 10.1038/s41746-026-02693-4

**Published:** 2026-04-28

**Authors:** Joel Troya, Ioannis Kafetzis, Ronja Weber, Yun Chiang, Venkatesh Parayitam, Philipp Sodmann, Dieter Ziegler, Frank Puppe, Andreas Nüchter, Alexander Meining, Alexander Hann

**Affiliations:** 1 Interventional and Experimental Endoscopy (InExEn), Internal Medicine II, University Hospital Würzburg, Würzburg, Germany; 2 Bavarian Cancer Research Center (BZKF), Würzburg, Germany; 3 Informatics XVII - Robotics, Julius-Maximilians-Universität, Würzburg, Germany; 4 Artificial Intelligence and Knowledge Systems, CAIDAS (Center for Artificial Intelligence and Data Science), Julius-Maximilians-Universität, Würzburg, Germany

**Keywords:** Computational biology and bioinformatics, Gastroenterology, Health care, Mathematics and computing, Medical research

## Abstract

Artificial intelligence (AI) is increasingly used in gastrointestinal endoscopy for polyp detection and classification. However, most AI models are trained on images from multiple video processors, whereas clinical environments typically rely on a single processor. We developed EndoStyle, a StarGANv2-based style transfer system trained to mimic the visual characteristics of five different endoscopic processors. On a validation dataset, Fréchet Inception Distance and Learned Perceptual Image Patch Similarity indicated high visual fidelity and perceptual similarity across processors. Semantic similarity analysis using three foundation models confirmed that converted images were equally consistent with both content and style inputs. In a multicenter study, endoscopists considered real and converted images realistic at comparable rates. When used to augment polyp detection model training, synthetic images significantly improved precision and specificity, reducing false positives by over 40% on two distinct evaluation datasets. EndoStyle thus offers a practical solution for processor-specific AI generalization.

## Introduction

Each year, over 17.7 million gastrointestinal (GI) endoscopic procedures are performed in the United States^1^. Artificial intelligence (AI), particularly computer vision, is becoming an integral part of the clinical routine^2,3^. The expeditious evolution of AI together with the ever-increasing volume of available data fosters the continuous development of innovative AI-based solutions^4,5^. These include, most notably, computer-aided polyp detection (CADe) and characterization systems^6–8^, but also systems for colonoscopy quality control^9^, automatic report generation^10,11^, and pathology grading^12,13^.

Despite its wide application, domain shift remains one of the most substantial obstacles in developing AI for colonoscopy^14,15^. Domain shift refers to differences between the distributions of the training and testing data of an AI model, which can be detrimental to its performance. It is known that different hardware, such as endoscopy processor and endoscope, and recording settings result in highly variable data appearance^16^. A recent work highlighted that differences in hardware result in different shape, location, and resolution of the endoscopic image^17^. Additional parameters where endoscopic images can differ are contrast, color depth, white balance, saturation, light intensity, dynamic range, and lens distortion. All parameters result in a significant shift when it comes to endoscopic data. This leads to commercially available AI systems being usually restricted to specific brands or hardware configurations and the underdevelopment of cross-hardware AIs^18^.

Domain shift has been observed in several medical fields and identified as a potential cause for limited AI generalizability^19,20^. It is commonly addressed via domain-invariant feature learning or advanced augmentation. Image style transfer, a technique initially developed for artistic purposes, has also found applications in medical imaging^21–23^. Shin et al. demonstrated that style transfer can improve the ability of deep learning models for digital pathology to generalize^24^. In radiology, style transfer technology has been used along with a conditional Generative Adversarial Network (GAN) to generate anatomically accurate, full-sized, computed-tomography images^25^. Lastly, in cardiovascular surgery, style transfer has been used to improve the realism of surgical simulators^23^. In colonoscopy, Zhang et al. explored the potential of a minimal GAN to improve polyp segmentation performance on multiple benchmark datasets^26^. Similarly, Golhar et al. used GAN inversion to synthesize realistic colonoscopy images for lesion classification^27^. However, to our knowledge, no study has yet explored the potential of style transfer techniques for improving polyp detection.

This work introduces EndoStyle, a novel AI-based system that performs image style transfer specifically for colonoscopy, aiming to mitigate the impact of domain shift during AI training. Images recorded with a given processor are considered to share common visual characteristics, which we define as the processor’s style. Given a colonoscopy image and a target style as input, EndoStyle converts the original image to match the target style while preserving original content. This capability can substantially enhance domain adaptation in colonoscopy, where each institution typically uses a specific hardware setup, resulting in locally acquired data that reflect a consistent style. EndoStyle can be applied to align training data with a target style by generating additional synthetic images that mimic the style of the intended hardware. This approach mirrors real clinical deployment scenarios, where a trained model must perform optimally on a known device.

Skepticism exists regarding the use of synthetic data in medical AI. To address this, images generated by EndoStyle were first validated for style fidelity using quantitative metrics and for content preservation through semantic similarity analyses between the content, style, and generated images. The plausibility of generated images was further assessed in a multi-center study in which participants were asked to determine whether images could originate from a given colonoscopy video, simultaneously evaluating both realism and accurate style transfer.

The potential of EndoStyle to improve AI performance was investigated in the context of polyp detection, a critical task in screening colonoscopy. The scenario in which an endoscopy clinic, using a specific processor, intended to develop a CADe system was considered. The available training data may come from two broad sources: multiple processors, including public datasets, multi-center studies, or clinic data spanning hardware changes; or a single processor, such as the one previously in use. To implement this scenario, two video datasets were used for testing, with all videos in each dataset recorded exclusively with a single processor. These represent the current processor of the clinic. For training, two cases were considered: one using images from public datasets spanning multiple processors, and another using only images from a single processor different from both test datasets. The impact of customizing the training dataset with synthetic images generated by EndoStyle to match the style of the target processor was evaluated. Particular attention was given to reducing false-positive detections, which could distract endoscopists and reduce clinical trust^28–30^.

## Results

### Evaluation of image generation

EndoStyle was trained with 239,875 images from 3452 colonoscopies recorded between 12 December 2018 and 18 March 2024 using 5 different endoscopic processors. The mean Fréchet Inception Distance (FID) between original and converted images was 27.2 and the mean Learned Perceptual Image Patch Similarity (LPIPS) score was 0.3 (Table 1).

**Table 1:** Image similarity metrics for style-transferred images across endoscopic processors

Source	Olympus	Olympus	Olympus	Pentax	Storz	Mean
	CV-1500	CV-190	CV-170	EPK-i7000	Image1 S	
FID	26.5	24.1	32.8	25.8	26.7	27.2
LPIPS	0.3	0.3	0.3	0.3	0.3	0.3

*FID* Fréchet Inception Distance, *LPIPS* Learned Perceptual Image Patch Similarity.

**Fig. 1:**
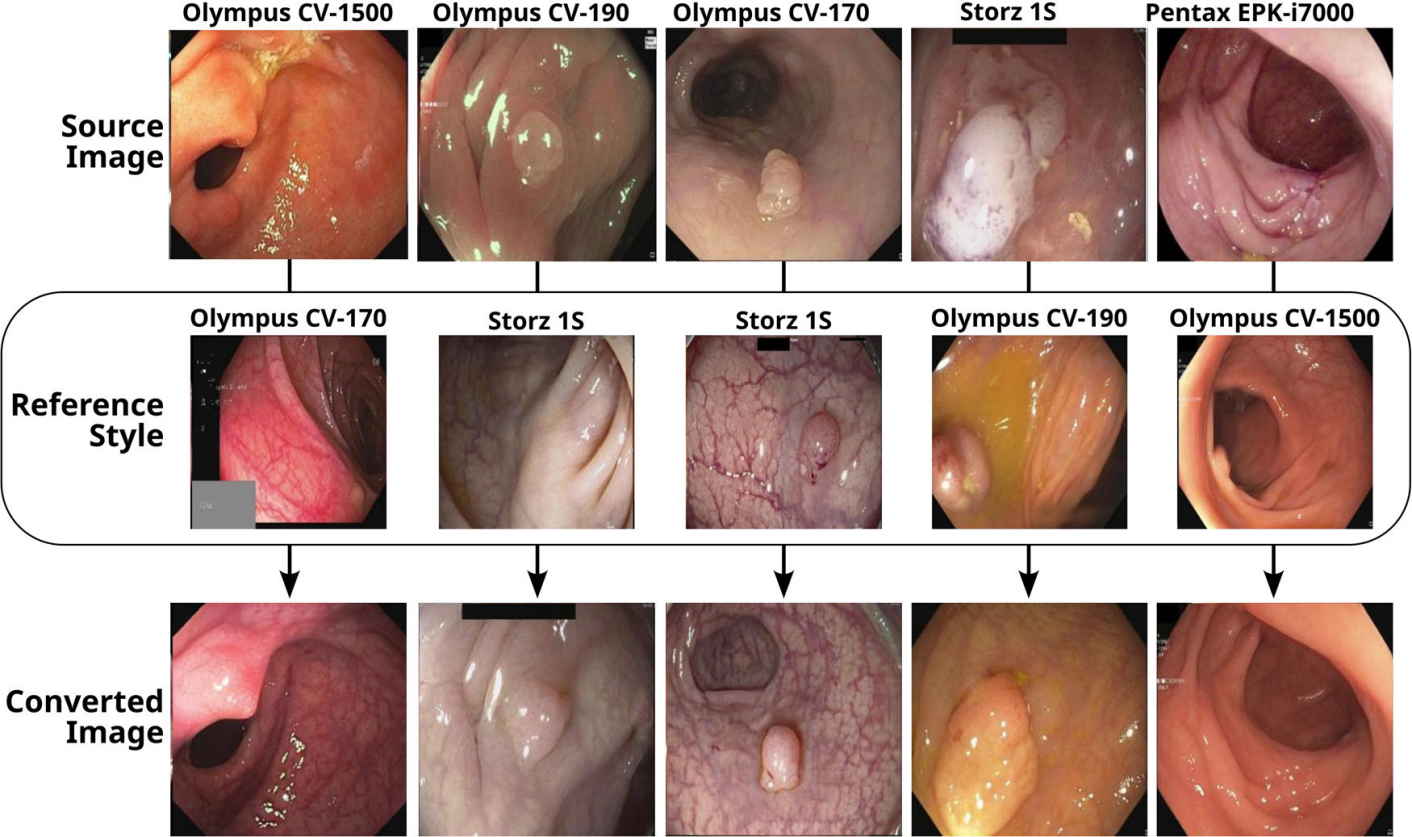
EndoStyle image style conversion examples. The first row corresponds to source images recorded with different processors. The second row to reference images recorded with a different processor than the source image. The third row contains the conversion of source image to reference style.

Examples of image style conversion are shown in Fig. 1. In each case, a source image and a reference (target style) image are used to generate a style-transferred image (converted image).

**Fig. 2:**
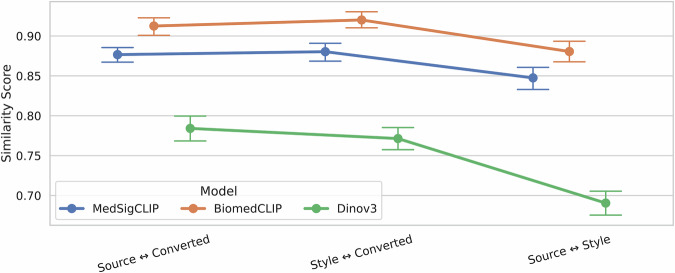
Semantic similarity between image pairs across three embedding models. Mean cosine similarity (±95% CI) between source and converted image pairs, style and converted image pairs, and source and style image pairs (*n* = 56).

Semantic similarity of the source, style and converted images are evaluated using similarity of features obtained from three different foundation models. Across all three models, the source–converted and style–converted similarity scores were consistently high and mutually comparable. For MedSigLIP, the source–converted mean similarity was 0.88 (95% CI: 0.87–0.89) and the style–converted mean was 0.88 (95% CI: 0.87–0.89). BiomedCLIP yielded higher overall scores, with source–converted similarity of 0.91 (95% CI: 0.90–0.92) and style–converted similarity of 0.92 (95% CI: 0.91–0.93). DINOv3 produced comparatively lower scores, with source–converted similarity of 0.78 (95% CI: 0.77–0.80) and style–converted similarity of 0.77 (95% CI: 0.76–0.79). In all three models, source–style similarity was significantly lower: 0.85 (95% CI: 0.83–0.86) for MedSigCLIP, 0.88 (95% CI: 0.87–0.89) for BiomedCLIP, and 0.69 (95% CI: 0.68–0.70) for DINOv3, with *p* < 0.001 in all cases (Fig. 2).

### Image realism evaluation

A total of 22 endoscopists from 16 different centers evaluated a total of 616 tasks including 1848 images. The distribution of evaluated images was 645 (34.9%) positive control (frames originating from the same colonoscopy), 596 (32.3%) negative control (frames not originating from the same colonoscopy), and 607 (32.8%) EndoStyle converted images. The background of the participating physicians as well as the experience with different video processors is described in Supplementary Table 1.

**Fig. 3:**
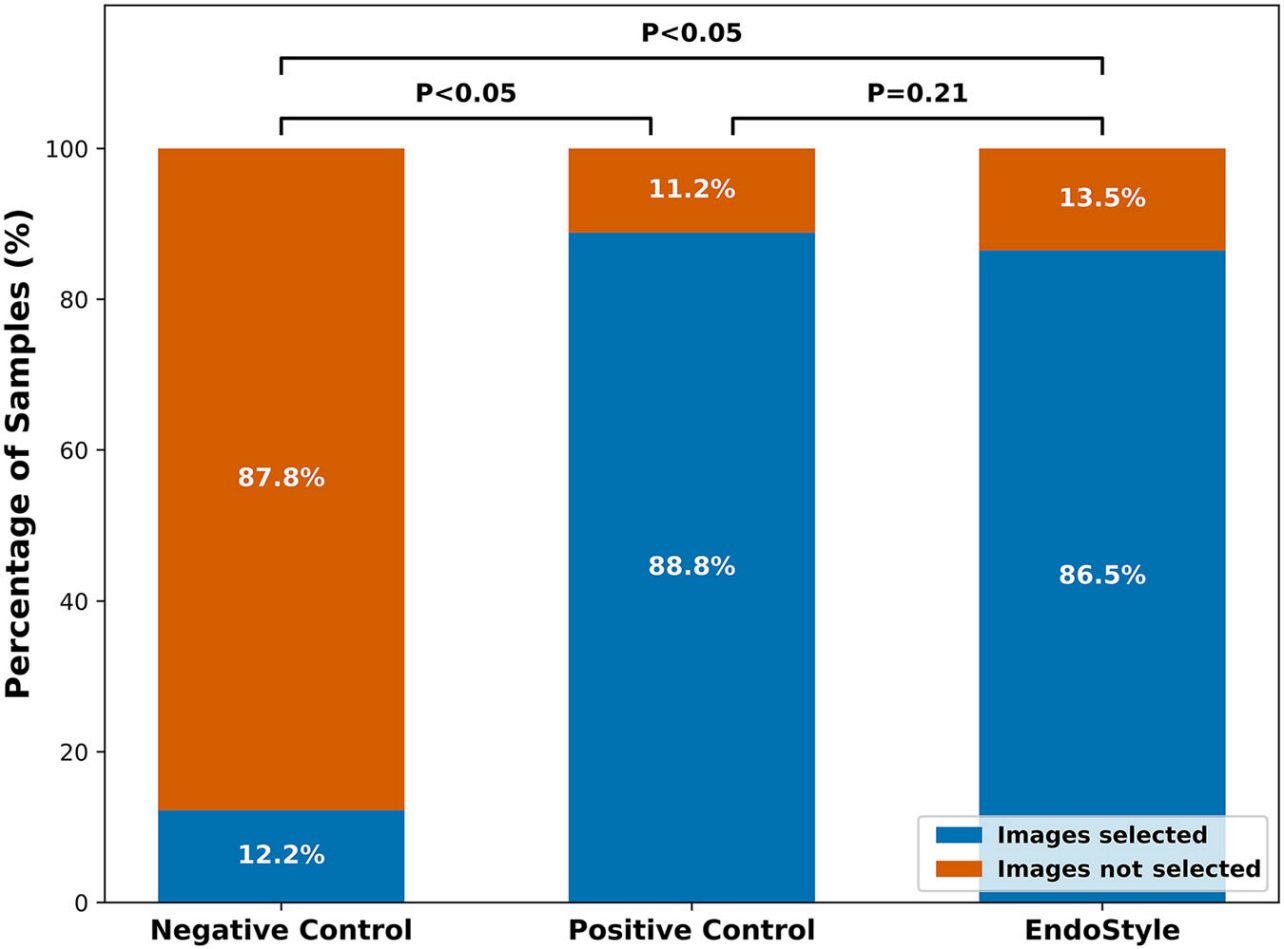
Stacked bar plot for image selection distribution in the image realism study. The plot shows the percentage of images identified as belonging to the inspected video sequence across negative control, positive control, and EndoStyle groups.

Endoscopists identified 573 (88.84%) positive control, 73 (12.25%) negative control, and 525 (86.49%) EndoStyle images as belonging to the same examination as the displayed video sequence. The distribution of selected images for each group of images is presented in Fig. 3. The proportion of images selected was significantly different between both positive-negative and endostyle-negative groups (*p* < 0.001). On the contrary, the proportion of selected images was comparable for the positive and EndoStyle groups (*p* = 0.206).

### Polyp detection with multi-source training data

For both test datasets, we evaluated models trained with 20% positive (containing at least one polyp) EndoStyle images and either 0, 20, 50, or 100% additional negative (without any polyps) data (Supplementary Table 4). Models trained with different percentages achieved similar results in terms of sensitivity and specificity (Supplementary Fig. 4). Therefore, for this study we analyze the models trained with 20% additional positive and 100% additional negative data, as larger training datasets are considered beneficial.

On Test Dataset 1, which comprises Olympus CV-190 data, the baseline model detected all polyps in at least one frame and achieved a per-frame sensitivity of 48.8%, specificity of 96.9%, and precision of 55.2%. The corresponding augmented model achieved sensitivity of 40.8% but specificity of 98.2% and precision of 63.7%. The result was a reduction of 17% for true positives (TP) and 43% of false positives (FP). In total, the model missed 5 flat, Paris IIa lesions^31^.

In Test Dataset 2, containing data recorded with Olympus CV-1500, a similar pattern was observed. The baseline model that was not trained on any CV-1500 image achieved a per-frame sensitivity of 50.7%, specificity of 95.7%, and precision of 48.4%. Adding 20% additional positive and 100% additional negative EndoStyle data resulted in sensitivity of 44.4%, specificity of 97.4%, and precision of 58.1%. The overall reduction in TPs was 12% and for FPs was 41%. In total, only one lesion was missed by the EndoStyle augmented model.

For these models, the impact of synthetic data on model behavior was evaluated by analyzing the precision-recall area under the curve (PR-AUC). In both cases the augmented model achieved higher but comparable results. For Test Dataset 1 both curves followed the same shape, whereas the curves are different in shape for Test Dataset 2 (Supplementary Fig. 7). This indicates that for Test Dataset 2, usage of EndoStyle training data impacts the learned representation for the model. In addition, for both test datasets, the average precision of the EndoStyle augmented model was slightly superior.

**Fig. 4:**
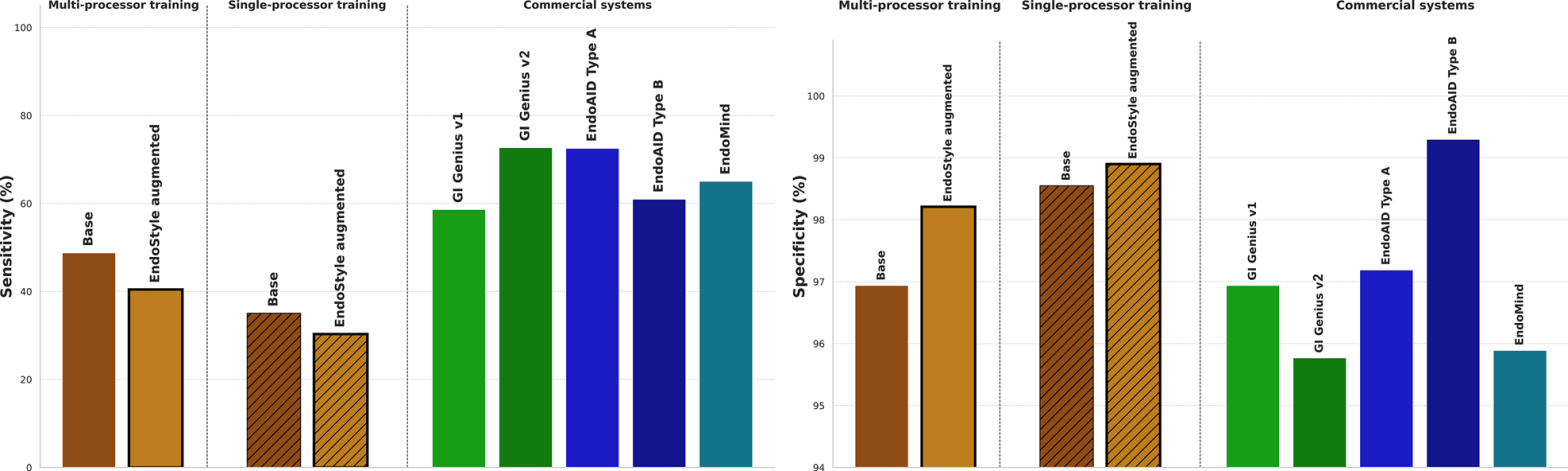
Comparison of the baseline and Endostyle augmented models with commercially available polyp detection systems. Models trained with data from multiple and single source training data are evaluated in terms of sensitivity (left) and specificity (right).

Comparison with commercially available AI-based polyp detection systems on Test Dataset 1 showed that sensitivities of both the baseline and EndoStyle models are lower than the commercially available systems, probably due to the low amount of data used to train them. However, the specificity of the EndoStyle models are superior to all the systems except EndoAID Type B (Fig. 4).

### Polyp detection with single, out-of-distribution processor data

Impact of EndoStyle was also evaluated in augmenting CADe training data recorded with Fujifilm processors (FUJIFILM Healthcare Europe GmbH, Ratingen, Germany), which were not included in the training of EndoStyle. On Test Dataset 1, the base Fujifilm model achieved a per-frame sensitivity of 35.1%, specificity of 98.6%, and precision of 65.2%. The corresponding augmented model achieved 30.3%, 98.9%, and 68.2%, representing a reduction in TPs of 13.2% and in FPs of 24.2%. In absolute terms, the base model missed 6 polyps, compared to 9 for the augmented model.

On Test Dataset 2, the base model yielded sensitivity, specificity, and precision of 38.5%, 98.1%, and 62%, respectively. The augmented model achieved 30.5%, 98.7%, and 65.5%, with a TP reduction of 21.6% and an FP reduction of 32.7%. Notably, neither the base model nor the augmented model missed any polyp, as at least one correct detection was achieved for each polyp in the Test Dataset 2.

Comparison with commercially available AI-based polyp detection systems on Test Dataset 1 showed that models achieve lower sensitivity compared to commercial ones, but higher specificity. Furthermore, models with data from multiple datasets are more sensitive than those trained with data from a single processor, which was out of distribution for EndoStyle (Fig. 4).

## Discussion

In this work, we introduce EndoStyle, a style transfer system that transforms colonoscopic images recorded with one processor to simulate visual characteristics of a different endoscopic processor. More explicitly, EndoStyle simulates acquisition with one of five different processors while preserving anatomical and pathological content. It consists of two components: a StarGANv2 model for low-resolution style transfer, and an enhanced deep residual network (EDRN) for image upscaling. A key contribution is the integration of two auxiliary AI-based losses during training. A depth-guided loss encourages preservation of structural features, such as the lumen and folds, while a polyp-segmentation loss promotes retention of true lesions and prevents the introduction of artificial polyp-like artifacts. Combination of the two losses is expected to enforce that any flat, subtle polyps present in the original image are also present in the generated one. EndoStyle was evaluated using: quantitative image quality metrics; semantic similarity of content, style, and converted images; and a multicenter realism study. Furthermore, its impact on training AI models for colorectal polyp detection in two realistic clinical scenaria is investigated, with particular focus on cross-domain generalization and the reduction of FP detections, a key barrier to clinical adoption of CADe systems.

Quantitatively, EndoStyle achieved a mean FID of 27.2 between real and generated images and a mean LPIPS of 0.3 across all five processors on a validation dataset, indicating high visual fidelity. Semantic similarities between converted–content and converted–style image pairs were high and statistically comparable across three models (MedSigLIP: 0.877 vs. 0.880; BiomedCLIP: 0.913 vs. 0.920; DINOv3: 0.784 vs. 0.771), and in all cases significantly higher than the content–style baseline (*p* < 0.001). The higher absolute similarities observed for BiomedCLIP and MedSigLIP compared to DINOv3 reflect the greater sensitivity of domain-adapted models to clinically relevant endoscopic features, and the convergence of results across all three independent embedding models strengthens confidence that the pipeline achieves meaningful stylistic adaptation without compromising anatomical content.

Subsequently, a multicenter study with 22 participants evaluated realism, style consistency, and content preservation In this, EndoStyle and positive control images had similar selection ratios (87% and 89% respectively), indicating that EndoStyle-generated images were perceived as realistic and stylistically consistent with true examination frames. The majority of participating endoscopists were highly experienced, with 19 out of 22 (86.4%) having performed more than 1000 colonoscopies. Therefore, our data did not support the investigation of the correlation between experience and perception.

While perceptual realism is important for clinical plausibility, it does not necessarily translate into improved domain alignment for deep learning models, which rely on distinct feature representations. Therefore, after establishing the quality and validity of the generated images, the potential of EndoStyle to enhance AI training for colorectal polyp detection was evaluated within a clinically relevant scenario. Specifically, we consider an endoscopy clinic using a single processor that aims to develop a CADe system optimized for that device, with training data originating either from multiple processors, such as public datasets or previous multi-center studies, or from a single processor, such as one previously used in the clinic.

Both cases were simulated. In the first, publicly available polyp images acquired with multiple processors were used. In the second, only images recorded with Fujifilm processor were selected from public datasets. Notably, in this latter case, EndoStyle had not been exposed to images from this processor during training. To approximate clinical deployment, two testing datasets of frame-level annotated colonoscopy videos were used, each recorded exclusively with a single processor, namely the Olympus CV-190 and the Olympus CV-1500. For the CV-190 dataset, benchmark performance from multiple commercially available systems was available from a previous study^32^. Importantly, images from the CV-1500 processor were entirely absent from the CADe training data in both scenarios.

In the first case, where training images were recorded with multiple processors, detection models were trained with varying proportions of EndoStyle-generated data. Synthetic augmentation consisted of a fixed proportion of polyp-containing images and increasing proportions of non-polyp images. The inclusion of negative samples specifically targeted the reduction of FP detections, a key barrier to the clinical adoption of CADe systems^32–35^. Incorporating EndoStyle-generated non-polyp images consistently improved specificity, indicating that processor-matched appearance information contributes to FP reduction, but at the expense of sensitivity. The comparatively lower sensitivity is likely attributable to the limited and heterogeneous training dataset, the scarcity of polyp examples, and the increased proportion of lesion-free frames.

An important observation is that the reduction in sensitivity remained stable across increasing proportions of synthetic non-polyp images, suggesting that EndoStyle does not introduce polyp-like artifacts during style transfer. If such artifacts were present, the absence of re-annotation would lead to mislabeled synthetic images containing unannotated polyps, progressively degrading sensitivity as their number increased. Instead, the additional missed polyps were primarily lesions already associated with low detection rate in the baseline model and in some cases the commercially available systems. This likely reflects their underrepresentation in the training data, an effect amplified by the addition of lesion-free images. Although it could be argued that style transfer might smooth subtle structural details characteristic of flat lesions, this was not supported by manual inspection of the generated images.

Given the comparable sensitivity and specificity trade-offs across configurations, the model trained with the largest dataset, comprising 100% EndoStyle-generated negative images in addition to the original positive samples, was selected for evaluation. This model achieved a sensitivity of 40.8%, specificity of 98.2%, and a reduction of 43% in FPs compared to the baseline model trained without EndoStyle augmentation for Test Dataset 1. For Test Dataset 2, sensitivity and specificity were 50.7% and 95.7% respectively, and the FP reduction 41%. Analysis of the PR-AUC showed that the average precision is slightly better in both test datasets for the EndoStyle model. The curves of the Baseline and the EndoStyle model largely overlap in Test Dataset 1, suggesting that the dominant effect in this case is an operating point shift. Nevertheless, in Test Dataset 2, the EndoStyle model yields a different curve shape reflecting a change in the underlying confidence ranking of detections.

When training was restricted to data from a single processor (Fujifilm), which was not seen during EndoStyle training, the model was augmented with 20% positive and 100% negative EndoStyle-generated data, based on prior experiments. The augmented model achieved a sensitivity of 35.1% and specificity of 98.6%, with an FP reduction of 24.2% in Test Dataset 1. For the second Test Dataset, sensitivity was 30.5%, specificity 98.7% and the FP reduction was 32.7%. These findings are consistent with those observed in the multi-processor setting. Due to limited data availability, this dataset was smaller than in the multi-processor scenario, which could also have contributed to the reduced sensitivity.

Domain shift is a common challenge in medical computer vision. Traditional image augmentation techniques^27,36^, such as rotation or noise injection, cannot reproduce the characteristic styles of different acquisition devices. Fine-tuning models using data from the target distribution are effective but require additional annotated data, which may be difficult to obtain for newer or less common hardware. EndoStyle provides an alternative by enabling processor-specific adaptation of existing datasets without requiring new annotations.

Previous work on style transfer and domain adaptation in gastrointestinal endoscopy has primarily focused on synthetic-to-real translation, visual enhancement, tissue simulation, or depth estimation. For example, Mahmood et al.^37^ proposed reverse domain adaptation to enable depth prediction from synthetic data, while Resindra et al. generated virtual chromoendoscopy images for 3D reconstruction^38^. Furthermore, Zhang et al.^26^ and Golhar et al.^27^ demonstrate that synthetic images generated via style transfer or GAN inversion can improve segmentation and classification performance. EndoStyle addresses real-to-real style transfer between endoscopic video processors to address the domain shift introduced. Consistent with prior studies, our results suggest that processor-specific synthetic data can also improve polyp detection.

This study has several limitations. First, EndoStyle was developed and evaluated using a limited number of endoscopic video processors, and its ability to generalize to newer devices remains to be validated. Next, the realism assessment was performed using still images rather than video sequences. Because EndoStyle operates on individual frames, applying it to sequential video data may introduce temporal inconsistencies and motion artifacts. Additionally, although the integration of depth-guided and segmentation-guided losses substantially reduced unrealistic features such as artificial lesions or flattened luminal structures, no dedicated ablation study was conducted to quantify the individual contributions of these losses, primarily due to the practical constraints of repeated expert evaluations. Considering the impact of EndoStyle on polyp detection performance, evaluation was performed using only two datasets consisting of a relatively small number of annotated videos, both recorded with Olympus hardware, as these were readily available. Further validation using datasets from additional manufacturers is required. Finally, fixed ratios of synthetic images with and without polyps were used to systematically analyze their influence on detection performance. Future work could explore adaptive sampling strategies, such as focusing synthetic data generation on uncertain or borderline cases or incorporating human-in-the-loop approaches to dynamically balance sensitivity and specificity while preserving clinical interpretability.

EndoStyle can serve as a foundation for further research on style transfer in colonoscopy. By adapting existing datasets to new acquisition settings, EndoStyle extends the utility of heterogeneous or legacy data for training modern AI systems. In the current implementation, vessel patterns are treated as part of image style rather than content. While selecting reference images can influence the transferred appearance, explicitly decoupling vascular structures from style represents an important direction for future work. Additionally, developing automated methods to verify content consistency between original and generated images, ideally with provable guarantees, would further strengthen clinical applicability. Of course, exploring alternative style transfer approaches, including diffusion-based models, and developing methods that eliminate the need for paired training data are additional avenues for future research. Further studies could also compare style transfer with fine-tuning when limited target-domain data are available. In this experiment, Fujifilm images were used exclusively as content inputs. The inverse scenario, that is, using Fujifilm images as the style reference, was not evaluated, as it falls outside the scope of this work and is unlikely to yield meaningful improvements given EndoStyle’s training distribution.

Overall, this study demonstrates that EndoStyle can generate realistic, processor-specific synthetic images and highlights its potential clinical utility. By adapting training data to the imaging characteristics of a target processor, EndoStyle offers a practical approach to improving CADe specificity and reducing FPs. Beyond diagnostic applications, style transfer may also support endoscopy training by enabling more realistic educational simulators.

## Methods

### Architecture and training of EndoStyle

**Fig. 5:**
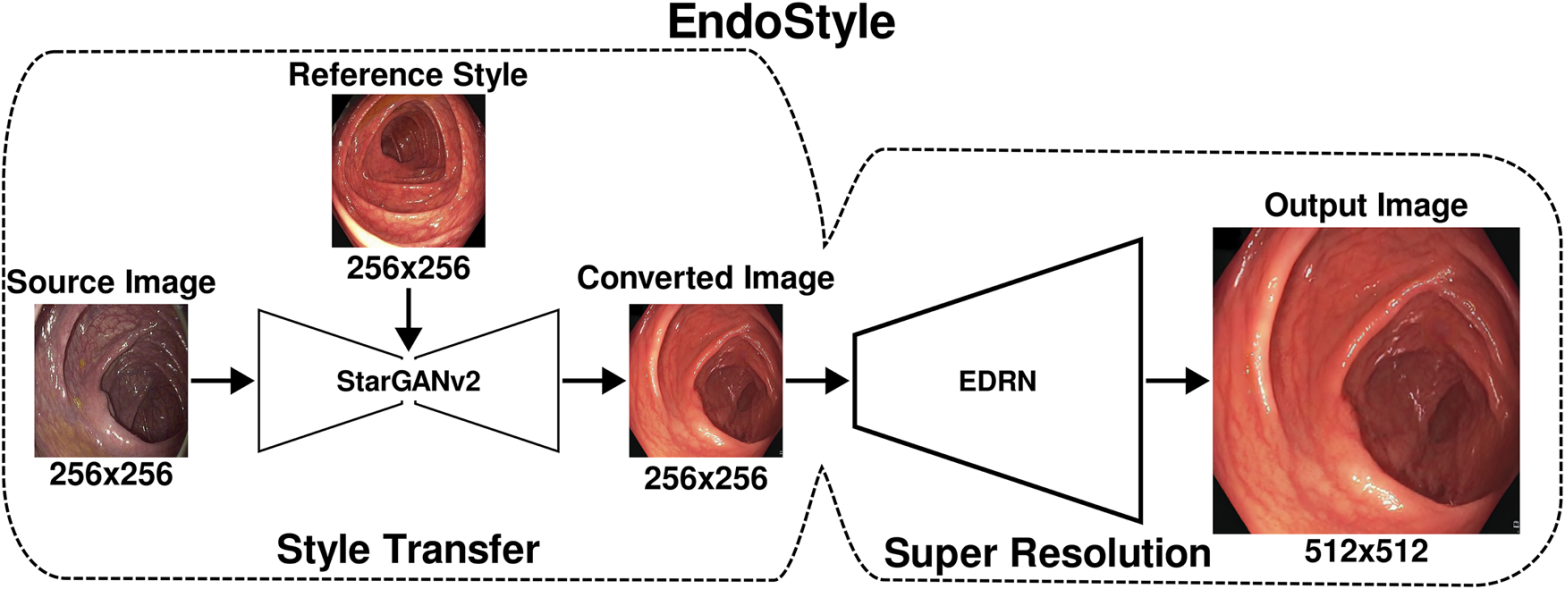
The EndoStyle system. The source image and reference style serve as input to the style transfer component (StarGANv2). The converted image, which serves as intermediate output, is passed through a super-resolution model (Enhanced Deep Residual Network (EDRN)) to obtain the output image. Image resolution is displayed under each image.

EndoStyle consisted of two AIs, one module responsible for style transfer and a second one for upscaling the image into a higher resolution. For style transfer, the StarGANv2^39^ model was selected, due to its potential to separate the content and style of an image for multiple styles. Additionally, StarGANv2 does not require paired data. The resolution of the images generated from the style transfer module was 256 × 256, which was found insufficient upon internal validation during development. To upscale the images, an Enhanced Deep Residual Network (EDRN)^40^ for super resolution was trained, with the same training and validation split as StarGANv2. The input to the model was a 256 × 256 endoscopic image, and the output was 512 × 512, that is, double the resolution image. The EDRN was applied directly to the output of the StarGANv2, to obtain the final output of EndoStyle. All synthetic images presented in the subsequent downstream evaluations had a uniform resolution of 512 × 512. The entire pipeline for style adaptation is shown in Fig. 5.

To train the model, data were acquired using five different endoscopic video processors: CV-170, CV-190, CV-1500 (Olympus Europe, Germany), Image1 S (Karl Storz SE, Germany), and EPK-i7000 (Pentax Europe GmbH, Germany). A 90–10 train-validation split on patient level was used to prevent data leakage during training. All training data consists of video frames from colonoscopy examination recordings, meaning that no paired data was available. The training data contained images recorded with both white light and advanced imaging. For each available examination, 1 in every 100 frames was sampled to mitigate the inclusion of similar images. Subsequently, a multi-classification AI assessed the quality of the images, excluding all low-quality images from the training dataset^8^. As part of image pre-processing, the endoscopic image region corresponding to the endoscope camera signal was automatically extracted using an AI-based method that excludes any graphical overlay or metadata regions^19^. Furthermore, all images were resized to 256 × 256 and normalized per color channel for training of the style transfer model. During training, class imbalance was handled by sub-sampling an equal number of examples for each processor.

Regarding style transfer, preliminary experiments using the baseline StarGANv2 framework revealed visual artifacts in the generated images. First, the generated images were not always consistent in terms of depth with the original images. Second, some polyps, mostly flat ones, were missing from the generated images, as the network could not separate them from the surrounding mucosa. To mitigate these issues, two additional losses were included in the training, namely depth and polyp loss. For the first loss, the depth mask was calculated using the MiDaS model^41^. The mean absolute difference between the two masks was calculated and added to the loss of the generator. Similarly, an available AI for polyp segmentation was employed to obtain binary masks indicating the location of polyps in both the original and converted image. The dice loss between the two masks was also added to the generator loss, to enforce consistency in polyp generation. Details about training hyperparameters and the implementation of these losses are given in the *Supplementary Data*.

### Image realism evaluation

The first objective was to assess the quality and realism of synthetic images generated with EndoStyle. These were assessed on the validation dataset using the FID, which quantifies the similarity between the distribution of real and generated images, and LPIPS, which measures perceptual similarity between image pairs, with lower values indicating better performance for both metrics^42,43^. Cycle consistency, that is, converting an image to a different style and then back to the original one, was also monitored^44^.

To evaluate the semantic fidelity of the style transfer pipeline, we assessed pairwise cosine similarity between image embeddings across three pairs: source–converted, style–converted, and source–style. This analysis was conducted on 56 triplets, each consisting of a source (content) image, a style image, and the corresponding converted image. Embeddings were extracted using three independent models: MedSigLIP^45^ and BiomedCLIP^46^, both domain-adapted vision-language models trained on medical imaging data, and DINOv3^47^, a general-purpose self-supervised vision transformer. For each model and each image pair, mean cosine similarity and 95% bootstrap confidence intervals were computed across all 56 triplets.

### Image realism experiment

Additionally, we conducted a user study in which endoscopists with varying levels of expertise evaluated 28 ten-second colonoscopy video sequences, each paired with three still images. Their task was to select any images they believed originated from the same recording as the video sequence. An example of the study interface is shown in Supplementary Fig. 1. For each video, three images were presented, which could belong to any combination of the three groups: positive control (images from the same recording as the displayed video), negative control (images from a different endoscopic processor), and EndoStyle (synthetic images originally recorded with a different processor and transformed to match the style of the displayed sequence). The images were randomized to ensure balanced exposure to each group. Each participant was randomly assigned a task for each video, so any overlap in annotated images was incidental. Consequently, inter-rater agreement could not be calculated, and the reported percentages correspond to the average across all rates.

It was assumed that experienced endoscopists would be able to recognize images originating from the same recording as the presented video clip and distinguish them from images that did not. Accordingly, if EndoStyle generated realistic images with appropriate style transfer, these images would be selected as belonging to the same recording at a frequency comparable to that of real images. Formally, it was hypothesized that images transformed with EndoStyle could not be reliably distinguished from positive control images, resulting in similar selection rates for both groups. In contrast, images from the negative control group were expected to be selected significantly less frequently than images from the other two groups.

### Synthetic data impact with multi-source training data

The second objective was to determine whether synthetic data generated by EndoStyle could enhance the performance of AI-based polyp detection, one of the most prominent applications of AI in colonoscopy^48^. To this aim, we trained multiple CADe systems with different proportions of synthetic data. We collected images from publicly available datasets that served as the baseline to create a Base Model. Subsequently, multiple “Augmented” models were developed, each trained on the same baseline data supplemented with additional EndoStyle transformed data.

#### Polyp detection training and testing data

Publicly available polyp and non-polyp images from three different datasets, the SUN^49^, HyperKvasir^16^, and PolypDB^50^, were collected (Supplementary Fig. 3). Images of the SUN database were recorded using Olympus CF-HQ290ZI and CF-H290ECI (Olympus, Tokyo, Japan). The HyperKvasir dataset includes images acquired with standard endoscopy equipment from Olympus (Olympus Europe, Germany) and Pentax (Pentax Medical Europe, Germany). The PolypDB dataset comprises images captured using endoscopy systems from Fujifilm, Olympus, and Pentax, primarily with white light imaging (WLI), but also with various digital image enhancement techniques such as blue light imaging (BLI), flexible imaging color enhancement (FICE), linked color imaging (LCI), and narrow band imaging (NBI)^50^. A summary of datasets used for the whole study can be found in the Supplementary Table 2.

The Baseline Training Dataset used for the Baseline model consisted of 7330 images from these datasets. Polyp images were collected from the HyperKvasir and PolypDB datasets, while non-polyp images were obtained from the SUN database. Because the HyperKvasir dataset does not provide information regarding individual examinations, the data were divided on an image level using an approximately 90–10 training–validation split, resulting in 896 images for training and 104 for validation. The PolypDB dataset likewise lacks examination identifiers. From a total of 3934 images, 3530 were used for training and 400 for validation. Four images were excluded due to discrepancies between the original image size and the corresponding polyp binary mask used to extract bounding boxes. For the SUN database, which provides examination-level metadata, the split was performed on an examination level to prevent data leakage across sets. Only videos categorized as negative (i.e., without visible polyps) were selected, and frames were sampled at a rate of 1 frame per second to avoid duplicates. To maintain class balance, the number of negative samples was limited to approximately 50% of the positive data, yielding 2400 non-polyp images in total, of which 1800 were used for training and 600 for validation. This partitioning strategy ensured non-overlapping data between training and validation sets and maintained a realistic positive-to-negative ratio representative of clinical practice.

Testing was performed on two distinct in-house colonoscopy video datasets, hereafter referred to as Test Dataset 1 and Test Dataset 2. In both datasets, all video frames were manually annotated with bounding boxes indicating the presence and location of polyps. Test Dataset 1 comprised 101 full-colonoscopy frame-by-frame annotated videos from a prospective study^32^, 45 of which contained a total of 93 polyps. All colonoscopies in this dataset were recorded using the Olympus CV-190 video processor, and the dataset was described in detail previously^32^. Test Dataset 2 consisted of 12 full-colonoscopy frame-by-frame annotated videos from a prospective study (NCT06094270), including a total of 24 polyps. Unlike the first dataset, these colonoscopies were recorded using the newest Olympus endoscopy processor CV-1500.

#### Polyp detection architecture and training

All detection models were based YOLOv11‑nano (YOLO11n) architecture^51^, a one‑stage convolutional detector for automatic polyp localization. The optimization was performed with stochastic gradient descent, starting from an initial learning rate of 0.01, which followed a cosine annealing schedule with a short warm‑up phase at the beginning of training. To improve robustness and reduce overfitting, we used the built‑in YOLO data augmentation pipeline, including random horizontal flips, random scaling and cropping, small translations/rotations, and mild color jitter (changes in brightness, contrast, and saturation), while preserving the anatomical orientation of the endoscopy frames. Training was perfomed using the Ultralytics^52^ python library. Detection models were trained with varying amounts of additional synthetic data. Specifically, portions of the Training dataset were transformed using EndoStyle to match the visual style of the target endoscopic processors, that is, CV-190 for Test Dataset 1 and CV-1500 for Test Dataset 2.

CADe systems are typically characterized by high sensitivity but often suffer from excessive FP alerts^29,32^. To address this, our training design emphasized improving specificity by exposing the model to a broader variety of non-polyp examples that match the visual characteristics of the target processor. A fixed 20% proportion of style-transferred polyp images was across all augmented models, to ensure comparability. After style transfer, positive images were verified to still contain polyps consistent with the original bounding boxes, without performing image re-annotation. In contrast, the proportion of style-transferred non-polyp images was varied (20%, 50%, 100%) to investigate how the inclusion of increasingly diverse non-polyp examples affects the sensitivity–specificity balance. In total, five models were trained per test dataset, as detailed in Supplementary Table 3. The converted images extended the training dataset, without replacing the original images. Entries with type “Baseline” models were trained exclusively on original datasets. All images obtained via EndoStyle were transformed to white light and not advanced imaging.

#### Polyp detection models evaluation

All models were evaluated in a frame-by-frame manner across both test datasets. Ground-truth annotations consisted of frame-level labels (polyp/non-polyp) and bounding boxes indicating the spatial location of each visible polyp. For each frame, model predictions were compared against the ground truth to determine TPs), FPs, true negatives (TN), and false negatives (FN). These values were subsequently used to compute sensitivity, specificity, precision, F1-score, negative predictive value, positive predictive value, AUC, and precision-recall area under the curve (PR-AUC).

In accordance to the definitions in Brand et al.^53^, a model prediction was considered TP if a predicted bounding box overlapped with a ground-truth polyp bounding box, defined by an intersection-over-union (IoU) greater than 0. Predictions with IoU equal to 0 were classified as FPs. Ground-truth polyps for which no overlapping predicted bounding box was detected were counted as FN. Frames without ground-truth polyps and without predicted bounding boxes were considered TN. FP were counted per predicted bounding box, meaning that multiple FP detections within a single frame were counted individually.

In addition, in Test Dataset 1, we compared the performance of the five developed models with that of several commercially available or open-source AI-based polyp detection systems. These included two software versions of GI Genius (Medtornic, Ireland): version 1.0 (current as of March 2020, referred to as v1) and version 2.0.1 (current as of October 2021, referred to as v2); two detection modes of EndoAID (Olympus Europe, Germany; March 2022); and the in-house developed system EndoMind^8^.

### Synthetic data impact on AI polyp detection with a single out-of-distribution processor

Building on the performance gains demonstrated above, we designed an experiment to evaluate EndoStyle’s ability to bridge domain gaps arising from processor-level variation. To this end, we collected colonoscopy images acquired with Fujifilm processors from two public datasets: BKAI^54^ and REAL-Colon^55^. Critically, Fujifilm-processed images are entirely out-of-distribution for EndoStyle, as the model was never exposed to this processor family during training. The resulting dataset is also notably limited in scale compared to the multi-source dataset setting of the previous experiment, comprising 1295 polyp-positive images (77% sourced from BKAI) and 797 polyp-negative images (all from REAL-Colon). Three models were trained and evaluated in this setting. The base model was trained exclusively on this Fujifilm dataset, without any style augmentation. Two augmented models were then trained by supplementing the base dataset with EndoStyle-converted images, one targeting the Olympus CV-190 processor style and one targeting the Olympus CV-1500. In both augmented datasets, 20% of positive images were converted, while all negative images (100%) were converted as a ratio determined empirically from the results of the previous experiment. The CV-190-augmented and CV-1500-augmented models were evaluated on Test Dataset 1 and Test Dataset 2, respectively.

### Statistical analysis

To evaluate whether EndoStyle-transformed images were perceived as realistic by endoscopists, we analyzed the results of the image selection task using the chi-squared test. Selection frequencies of EndoStyle-transformed images were compared to those of positive control images, as well as those of negative control images.

To assess the impact of EndoStyle-generated synthetic data on the performance of the AI-based polyp detection models, model outputs were compared using McNemar’s test. The test was applied to paired frame-level prediction outcome to investigate the presence of statistically significant differences in the performance between models trained with and without EndoStyle augmentation. The tests were performed separately for each of the two test datasets. To directly compare specificity between different systems, non-parametric bootstrapping was used. A total of 10,000 resamples were drawn with replacement, and the specificity difference between models was calculated for each resample to obtain confidence intervals. To assess differences in similarity among pairs of content, style, and converted images, the Wilcoxon signed-rank tests were applied to each pairwise comparison, with a significance threshold of *p* < 0.05.

### Ethics

Prospective collection of endoscopic examinations during clinical routine was approved by the local ethical committee responsible for each study center (Ethik-Kommission Landesärztekammer Baden-Württemberg (F-2021–047, F-2020-158), Ethik-Kommission Landesärztekammer Hessen (2021–2531), Ethik-Kommission der Landesärztekammer Rheinland-Pfalz (2021–15,955), Ethik-Kommission University Hospital Würzburg (12/20, 20200114 04), and Ethik-Kommission University Hospital Würzburg (2021032901). All procedures were in accordance with the Helsinki Declaration of 1964 and later versions. Signed informed consent from each patient was obtained prior to participation.

## Supplementary information

Supplementary file 1_rev

## Data Availability

The datasets generated and/or analysed during the current study are not publicly available due to data protection policy of the institutions involved but are available from the corresponding author on reasonable request.
