# Supplementary material for: Gastrointestinal endoscopic image style transfer using EndoStyle to improve artificial intelligence prediction models
Source: NPJ Digit Med. 2026 Apr 28;9:340. doi: 10.1038/s41746-026-02693-4 (PMC13125597; doi:10.1038/s41746-026-02693-4)
Supplement: Supplementary file 1 — _rev [file 41746_2026_2693_MOESM1_ESM.docx]

# Supplementary Material

## Training of the style transfer AI

EndoStyle was implemented using the official StyleGANv2 repository (<https://github.com/clovaai/stargan-v2>) with two additional auxiliary losses for depth and polyp segmentation. During training, whenever style transfer was applied, depth and polyp segmentation masks were computed for both the original and transformed images. For depth, we used the pre-trained MiDaS model to obtain relative depth masks and calculated the L1 distance between the original and transformed masks, which was added to the loss to encourage preservation of scene geometry. For polyp segmentation, an internally developed model generated masks, and the Dice coefficient between the original and transformed masks was added to the loss, ensuring that polyps in the transformed images aligned with the original segmentation.

Training followed the standard StarGANv2 procedure on 256 × 256 images. Latent and hidden dimensions were set to 512, and the style dimension to 128. Cycle-consistency, style reconstruction, and regularization losses were each weighted by 1, as were the auxiliary depth and polyp segmentation losses. Training ran for 100,000 iterations with random cropping applied at a probability of 0.5. Batch size was 8 for both training and validation. The generator learning rate was 1e-4, while the mapping network and style encoder used 1e-6. The Adam optimizer was used with β1 = 0, β2 = 0.99, and a weight decay of 1e-4. Ten output images per domain were generated for evaluation. The high-pass filter was disabled during training.

All experiments were conducted on a single Linux Ubuntu 20.04 workstation with an NVIDIA GeForce RTX 3090 GPU, and total training time for the final model was approximately 23 hours.

## Image realism experiment

In order to assess the plausibility of generated images, as well as how physician felt that the generated images fit a provide context, a user study was conducted using an in-house developed online platform, named Lutetia. An example of the study interface is shown in **Supplementary Figure 1**. Each user was presented with a 10 second video from a colonoscopy (left) and three images (right). The user would then select any of the three images to belong in the same examination as the one where the 10 second clip was taken from. Each user was assigned 28 such tasks, to which they could navigate from a sidebar.


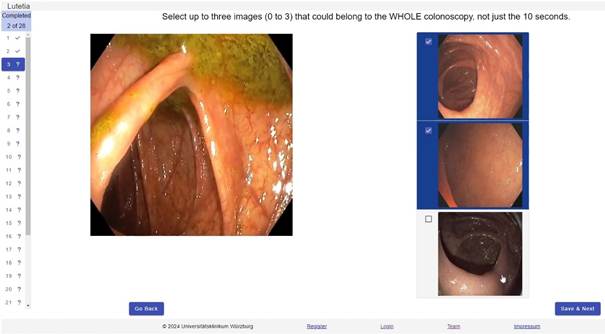


**Supplementary Figure 1**: Interface for the user study. Lutetia platform in which the image realism evaluation was conducted. On the left side of the screen, the colonoscopy video sequence was displayed and on the right side, endoscopists selected the images they believed could belong to the displayed video.

In total, 22 participants from 16 different institutions participated in the user study. The characteristics of the participants in the Lutetia study are presented in **Supplementary Table 1**.

**Supplementary Table 1**: Baseline characteristics of the participants in the EndoStyle image realism evaluation.

| **Center** | **Country** | **Number of participants** | **Gender** | | **Colonoscopy experience (procedures performed)** | | **Current videoprocessor used** | | |
| --- | --- | --- | --- | --- | --- | --- | --- | --- | --- |
|  |  |  | Male | Female | Between 400 and 1000 | More than 1000 | Olympus | Pentax | Fujifilm |
| Klinikum Stuttgart | Germany | 1 | 1 | 0 | 0 | 1 | 1 | 0 | 0 |
| Universitätsklinikum Aachen | Germany | 1 | 1 | 0 | 0 | 1 | 1 | 0 | 0 |
| St. Josef-Bochum | Germany | 1 | 1 | 0 | 0 | 1 | 1 | 0 | 0 |
| Universitätsklinikum Heidelberg | Germany | 2 | 2 | 0 | 1 | 1 | 2 | 0 | 0 |
| Universitätsklinikum Tübingen | Germany | 1 | 0 | 1 | 0 | 1 | 0 | 1 | 0 |
| DRK Kliniken Berlin | Germany | 1 | 0 | 1 | 1 | 0 | 1 | 0 | 0 |
| Universitätsklinikum Marburg | Germany | 1 | 1 | 0 | 0 | 1 | 0 | 0 | 1 |
| Niels-Stensen-Kliniken Osnabrück | Germany | 2 | 1 | 1 | 0 | 2 | 0 | 0 | 2 |
| Universitätsklinikum Bonn | Germany | 5 | 4 | 1 | 1 | 4 | 0 | 0 | 5 |
| Vivantes Humboldt-Klinikum Berlin | Germany | 1 | 1 | 0 | 0 | 1 | 1 | 0 | 0 |
| Katholisches Klinikum Mainz | Germany | 1 | 1 | 0 | 0 | 1 | 1 | 0 | 0 |
| Robert Bosch Krankenhaus Stuttgart | Germany | 1 | 1 | 0 | 0 | 1 | 1 | 0 | 0 |
| Universitätsklinikum Halle | Germany | 1 | 1 | 0 | 0 | 1 | 1 | 0 | 0 |
| Charite Berlin | Germany | 1 | 1 | 0 | 0 | 1 | 1 | 0 | 0 |
| University Hospitals Coventry and Warwickshire | United Kingdom | 1 | 1 | 0 | 0 | 1 | 1 | 0 | 0 |
| Sozialstiftung Bamberg Klinikum | Germany | 1 | 1 | 0 | 0 | 1 | 0 | 0 | 1 |
| Total |  | 22 | 18 | 4 | 3 | 19 | 12 | 1 | 9 |

In order to obtain insights, examples of correct and erroneous user assessments of images for all three image groups, along with a characteristic snapshot of the corresponding video clip are presented in **Supplementary Figure 2**.


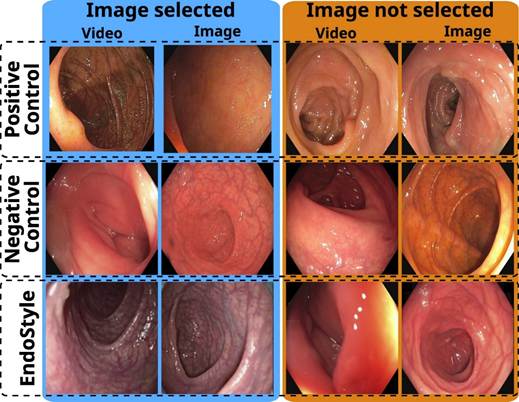


**Supplementary Figure 2:** Examples of selected and non-selected image pairs from the realism study. The left image shows a frame from the video clip presented to the endoscopist, while the right image shows the corresponding image evaluated as either belonging or not belonging to the same video recording.

## Polyp detection experiments

There were multiple datasets utilized in this work. For each of them, the source, hardware used in recording the data, and the role they played in the study is presented in **Supplementary Table 2**. It is worth noting that only a subset of the Real Colon dataset, recorded with Fujifilm, was considered in the study and thus described in this table. Furthermore, as the data in Real Colon are organized based on examination, the split was performed on examination and not image level to prevent data leakage.

**Supplementary Table 2**: Summary of datasets used for EndoStyle training and AI polyp detection model experiments. CADe: computer-aided polyp detection; WLI: White light imaging.

| **Dataset name** | **Role in study** | **Source / Center** | **Processor / Endoscope** | **Imaging mode** | **# images / videos** | **Split** |
| --- | --- | --- | --- | --- | --- | --- |
| **EndoStyle training set** | Style transfer training | 5 centers | Olympus CV-170, CV-190, CV-1500; Karl Storz Image1 S; Pentax EPK-i7000 | WLI + advanced imaging | 239,875 (3,452 colonoscopies) | 90/10 |
| **SUN** | CADe training (negative) | Public | Olympus CF-HQ290ZI and CF-H290ECI | WLI | 2400 | 75/25 |
| **HyperKvasir** | CADe training (positive) | Public | Olympus and Pentax | WLI | 1000 | 90/10 |
| **PolypDB** | CADe training (positive) | Public | Fujifilm, Olympus, Pentax | WLI + advanced imaging | 3930 | 90/10 |
| **BKAI** | CADe training  single-source (positive) | Public | Fujifilm | WLI | 1000 | 80/20 |
| **REAL-Colon** | CADe training  single source (positive & negative) | Public | Fujifilm | WLI | 1092 | 80/20 |
| **Test Dataset 1** | CADe test (external) | In-house | Olympus CV-190 | WLI | 101 videos (93 polyps) | - |
| **Test Dataset 2** | CADe test (external) | In-house | Olympus CV-1500 | WLI | 12 videos (24 polyps) | - |

## Polyp detection with Multi-Source Training Data

The first experiment conducted was training of a CADe system with data recorded with multiple processors. Training data was collected from three different publicly available datasets. The training data for the experiment are visually summarized in Supplementary Figure 2.


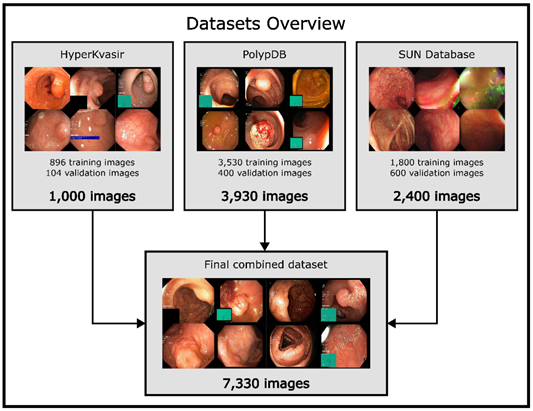


**Supplementary Figure 3**: Overview of the datasets for the mutli-source polyp detection system training. Public databases were used as a source to train and validate polyp detection models. In addition, part of the data was transformed using EndoStyle to expand the datasets.

Multiple detection systems were trained for both test datasets, using varying percentages of EndoStyle converted data. The different models considered in the evaluation are presented in **Supplementary Table 3**. Evaluation of the models in both datasets is presented in **Supplementary Table 4**.

**Supplementary Table 3**: Overview of training models, their configurations, and how they have been evaluated for the mutli-source training dataset.

| **Model** | **Type** | **Percentage of additional EndoStyle Non-polyp images** | **Percentage of additional EndoStyle polyp images** | **EndoStyle Targeted processor** | **Evaluated in dataset** |
| --- | --- | --- | --- | --- | --- |
| 1 | Baseline | 0 | 0 | CV-190 | Test Dataset 1 |
| 2 | Augmented | 0 | 20 | CV-190 | Test Dataset 1 |
| 3 | Augmented | 20 | 20 | CV-190 | Test Dataset 1 |
| 4 | Augmented | 50 | 20 | CV-190 | Test Dataset 1 |
| 5 | Augmented | 100 | 20 | CV-190 | Test Dataset 1 |
| 6 | Baseline | 0 | 0 | CV-1500 | Test Dataset 2 |
| 7 | Augmented | 0 | 20 | CV-1500 | Test Dataset 2 |
| 8 | Augmented | 20 | 20 | CV-1500 | Test Dataset 2 |
| 9 | Augmented | 50 | 20 | CV-1500 | Test Dataset 2 |
| 10 | Augmented | 100 | 20 | CV-1500 | Test Dataset 2 |

**Supplementary Table 4**: Evaluation results for the trained models on both validation datasets.

|  | **Model** | **Per-frame sensitivity** | **Per-frame specificity** | **Per-frame precision** | **Per-frame**  **F1 score** | **Negative predictive value** | **Positive predictive value** | **AUC** |
| --- | --- | --- | --- | --- | --- | --- | --- | --- |
| Test Dataset 1 | Baseline | 48.8% | 96.9% | 55.2% | 51.8% | 96.1% | 55.2% | 74.9% |
|  | 0% | 44.2% | 97.0% | 53,4% | 48.4% | 95.7% | 53.4% | 73.0% |
|  | 20% | 40.5% | 98.0% | 61.2% | 48.8% | 95.5% | 61.4% | 72.6% |
|  | 50% | 40.3% | 98.1% | 62.1% | 48.9% | 95.5% | 62.1% | 71.2% |
|  | 100% | 40.4% | 98.2% | 63.7% | 49.5% | 95.5% | 63.7% | 73.1% |
| Test Dataset 2 | Baseline | 50.7% | 95.7% | 48.4% | 49.5% | 96.0% | 48.4% | 91.4% |
|  | 0% | 45.2% | 96.9% | 53.9% | 49.2% | 95.7% | 53.9% | 88.4% |
|  | 20% | 44.5% | 97.1% | 55.6% | 49.4% | 95.6% | 55.6% | 90.1% |
|  | 50% | 44.8% | 97.5% | 59.3% | 51.1% | 95.6% | 59.3% | 88.0% |
|  | 100% | 44.4% | 97.4% | 58.1% | 50.3% | 95.6% | 58.1% | 89.9% |

When training with additional EndoStyle-transformed images, FP rates dropped by up to 41.2%, depending on the proportion of synthetic data used (20%, 50%, or 100%). This reduction came at the cost of a 17% drop in TPs, resulting in three, two, and five flat, Paris Classification IIa, polyps being missed for the models containing 20%, 50%, and 100% additional EndoStyle-transformed data, respectively. Despite the decrease in TPs, the overall model performance, measured by the per-frame precision, increased and reached up to 63.7%.

The percentage of TPs and FPs for all models corresponding to Test Dataset 1 and Test Dataset 2, as increasing numbers of EndoStyle-generated synthetic non-polyp images are added to the training, is presented in **Supplementary Figure 4**. The left panel corresponds to Test Dataset 1, whereas the right panel corresponds to Test Dataset 2.

**
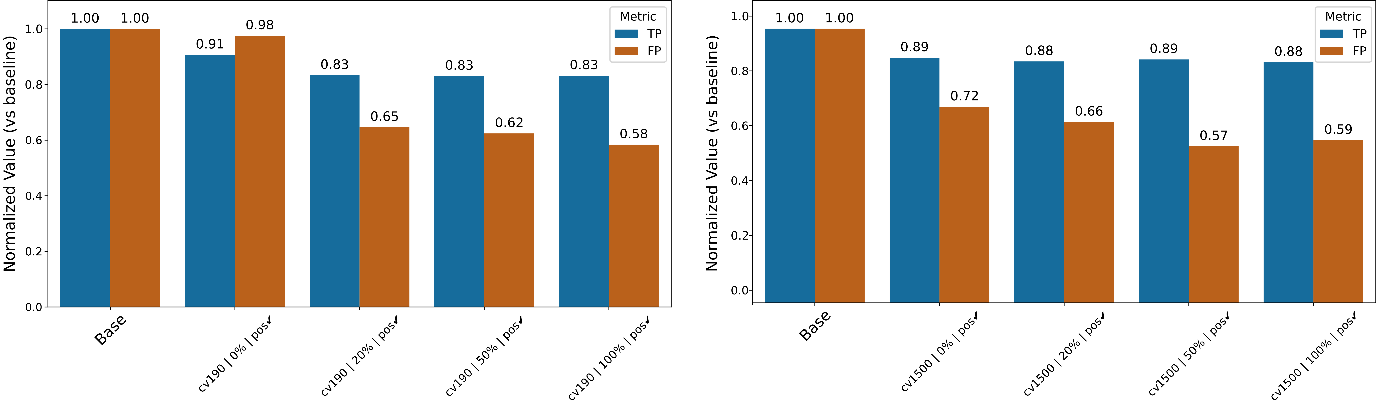
**

**Supplementary Figure 4**: Effect of EndoStyle-generated synthetic non-polyp images on detection performance. The left panel shows the performance of the different models in Test Dataset 1. The right panel shows the performance for Test Dataset 2. True positive and false positive counts are normalized to the baseline model (base=1.00). Values below 1.00 indicate a reduction relative to the baseline.

There were polyps in the test datasets that were missed by the polyp detection systems, and some also by commercially available CADe systems. Examples of such lesions are visualized in **Supplementary Figure 5**.


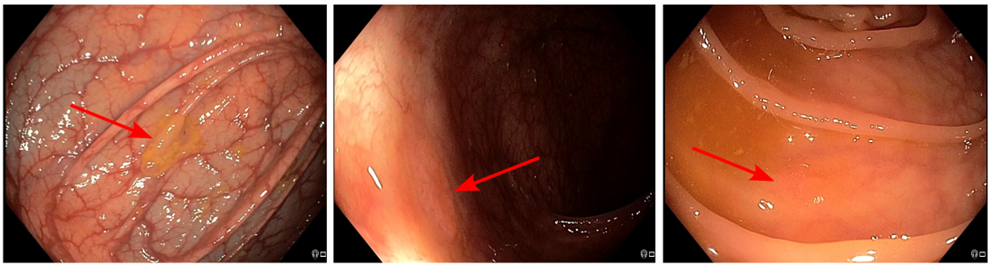


**Supplementary Figure 5:** Examples of lesions missed by the trained polyp detection systems. Red arrows indicate the location of the lesion in the image.

Comparison with commercially available AI-based polyp detection systems on Test Dataset 1 showed that the Baseline model and GI Genius v1 achieved similar specificity of 96.9%. While statistical analysis indicated a significant difference between them, the actual difference was below 0.004 percentage points, and thus unlikely to be clinically relevant. In contrast, incorporating EndoStyle-transformed images into the training set led to a significant and meaningful increase of specificity compared to all commercial systems, with the exception of EndoAID Type B (**Supplementary** **Figure 6 – right panel**). In terms of sensitivity, the Baseline model exhibited lower performance compared to all commercial systems. Inclusion of EndoStyle-transformed data in the training did also not increase the sensitivity (**Supplementary** **Figure 6 – left panel**).


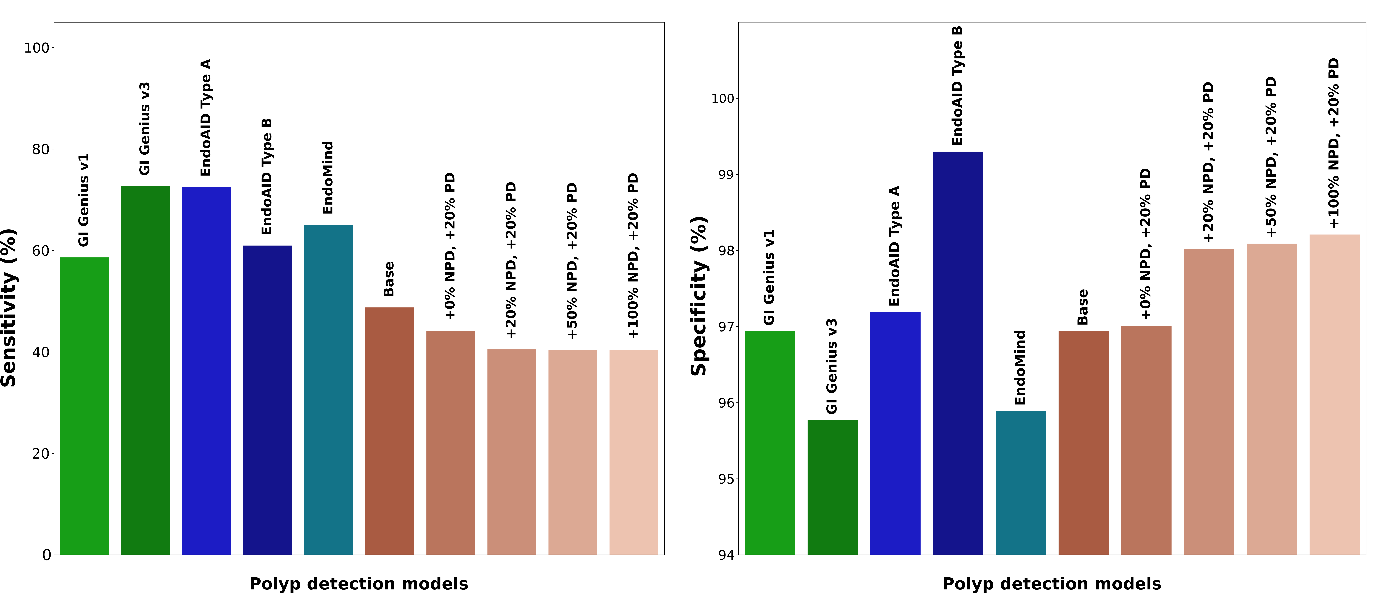
**Supplementary Figure 6**: Comparison of various polyp detection systems using Test Dataset 1. The left panel shows the sensitivity across different polyp detection models, including commercially available polyp detection systems. The right panel displays the corresponding specificity for the same models. Models labeled with 0% 20%, 50%, and 100% indicate the proportion of EndoStyle-transformed non-polyp images added to the original training set. NPD: Non-polyp data; PD: Polyp data.

Since performance remained comparable across different levels of negative data, models trained with 100% negative data were selected for subsequent analysis, as they incorporated the largest number of training samples.

To assess whether EndoStyle augmentation alters model behavior or merely shifts the operating point, we compared precision–recall curves rather than single-threshold metrics (**Supplementary Figure 7**). In Test Dataset 1, both models exhibit nearly identical curve shapes with only a marginal improvement in average precision, suggesting limited change in ranking behavior and a possible effect of threshold calibration. In contrast, in Test Dataset 2, the EndoStyle model consistently outperforms the baseline across a wide range of recall values, indicating a genuine improvement in the model’s discriminative ability rather than a simple shift in operating point.





**Supplementary Figure 7:** Precision-Recall curves and their area under the curve for the baseline and augmented models. The left panel corresponds to Test Dataset 1 and the right panel on Test Dataset 2. AP: Average Precision.
